# Roles of Tumor Markers in Central Nervous System Germ Cell Tumors Revisited with Histopathology-Proven Cases in a Large International Cohort

**DOI:** 10.3390/cancers14040979

**Published:** 2022-02-15

**Authors:** Hirokazu Takami, Christopher S. Graffeo, Avital Perry, Caterina Giannini, Yoichi Nakazato, Nobuhito Saito, Masao Matsutani, Ryo Nishikawa, Koichi Ichimura, David J. Daniels

**Affiliations:** 1 Department of Neurosurgery, Faculty of Medicine, The University of Tokyo, Tokyo 1138655, Japan; nsaito-nsu@m.u-tokyo.ac.jp; 2 Department of Neurologic Surgery, Mayo Clinic, Rochester, MN 55905, USA; graffeo.christopher@mayo.edu (C.S.G.); perry.avital@mayo.edu (A.P.); 3 Department of Laboratory Medicine and Pathology, Mayo Clinic, Rochester, MN 55905, USA; giannini.caterina@mayo.edu; 4 Department of Pathology, Hidaka Hospital, Gunma 3700001, Japan; nakazato_yoichi@gunma-u.ac.jp; 5 Division of Pediatric Neuro-Oncology, Saitama Medical University International Medical Center, Saitama 3501298, Japan; matutani@saitama-med.ac.jp (M.M.); rnishika@saitama-med.ac.jp (R.N.); 6 Department of Brain Disease Translational Research, Juntendo University Faculty of Medicine, Tokyo 1138421, Japan; k.ichimura.uk@juntendo.ac.jp

**Keywords:** germ cell tumor, alpha fetoprotein, human chorionic gonadotropin, tumor marker

## Abstract

**Simple Summary:**

The optimal cut-offs for tumor markers in CNS germ cell tumors (GCTs) to specify non-germinomatous GCTs are under active investigation. This study investigated 162 cases with histopathological confirmation and tumor markers, including 77 cases with robust specimens obtained from tumor resection. This study demonstrates that tumor markers are elevated in germinomas and teratomas, especially HCG in germinomas and AFP in immature teratomas, emphasizing the clinical significance of tissue diagnosis. Similarly, some biopsy cases without malignant findings on histopathology demonstrated tumor marker elevations, highlighting the limitations of reliable tissue diagnosis from a diminutive specimen. This study concludes that an integrated diagnosis based on tumor markers and histopathology is the key to precise diagnosis and, therefore, the avoidance of over- or under-treatment. This study also invites novel questions regarding whether marker-positive germinomas or teratomas should be treated as intensively as malignant NGGCTs, as well as the optimal treatment strategy for marker-negative immature teratomas.

**Abstract:**

The central nervous system germ cell tumor (CNS GCT) is a rare and incompletely understood disease. A major outstanding question in the 2015 consensus document for CNS GCT management was the utility and interpretation of the tumor markers human chorionic gonadotropin (HCG) and alpha fetoprotein (AFP) in the diagnosis of malignant non-germinomatous GCTs (hereafter NGGCTs) prior to treatment. In the current study, we assembled two geographically and ethnically different clinical cohorts from the Mayo Clinic (1988–2017) and the intracranial GCT Genome Analysis Consortium (iGCT Consortium) in Japan to address this question. Patients with both histopathological diagnosis and tumor markers available were eligible for inclusion (*n* = 162). Biopsy and surgical resection were performed in 85 and 77 cases, respectively. Among 77 resections, 35 demonstrated positivity for HCG, AFP, or both (45%). Seventeen of the marker-positive cases had no malignant non-germinomatous component identified on histopathology, but they were composed strictly of germinoma, teratoma, or both (49%). One embryonal carcinoma was the only marker-negative NGGCT in the study sample. Among 85 biopsies, 18 were marker positive (21%). Seven of these patients had no malignant non-germinomatous component on histopathology, suggesting the potential limitations of limited tissue sample volumes. Neither histopathological diagnosis nor tumor markers alone reliably diagnose NGGCTs due to the secretion of HCG and AFP by germinomas and teratomas. Treatment planning should incorporate integrated histopathological and laboratory-based diagnosis to optimize diagnostic and treatment strategies for this unusual and histologically heterogeneous tumor.

## 1. Introduction

Central nervous system germ cell tumors (CNS GCTs) are neoplasms, mainly occurring in pediatric, adolescent, and young adult populations, and they encompass several distinct histopathological entities. The most frequent histopathological variant is germinoma, which comprises more than half of the cases [1]. Non-germinomatous GCTs (NGGCTs) in a broad sense consist of mature/immature teratoma (MT/ImT), teratoma with malignant transformation, yolk sac tumor (YST), embryonal carcinoma (EC), and choriocarcinoma (CC) [2]. However, many GCTs have multiple histological components in one tumor, and they are called mixed GCTs. GCTs usually occur in midline locations, most often in the pineal gland, followed by the neurohypophysis—often in tandem, known as bifocal tumors [3,4,5,6,7].

Tumor markers (human chorionic gonadotropin (HCG) and alpha fetoprotein (AFP)) in serum and cerebrospinal fluid (CSF) are useful for diagnosis [8], the evaluation of treatment response [9], and detecting recurrence after treatment [10]. GCTs have been shown to transform histologically following treatment, with associated fluctuations in tumor markers [11]. Although the definitive diagnosis of GCTs requires tissue, in the setting of elevated tumor markers, imaging features, and clinical findings, histopathological diagnosis may be omitted prior to treatment induction [12].

Conceptually, this is motivated by the fact that most cases demonstrating elevated tumor markers are NGGCTs with a malignant component (YST, EC, CC, and malignant transformation of teratoma, referred to as NGGCTs hereafter). However, it is generally accepted that treatment without histopathologic diagnosis exchanges procedural risk for a potentially non-indicated and toxic treatment, resulting in disagreement regarding the optimal tumor marker cut-off for initiating treatment without tissue diagnosis [12]. This landscape is further complicated by marker-positive germinoma cases [13], including a number of previously reported germinoma cases with HCG elevation, suggesting that the conventional presumption may over-estimate NGGCT prevalence. For example, a bifocal tumor at the neurohypophysis and pineal gland presenting with diabetes insipidus and negative tumor markers has traditionally been assumed to be germinoma; however, contemporary cohort studies indicate a prevalence of at least 3.4% NGGCTs in this bifocal CNS GCT subpopulation [14]. In contrast, histopathological diagnosis has inherent procedural risks, as well as the risk of inadequate or inaccurate sampling yielding a false-negative result. Although tissue diagnosis is considered the gold standard at most centers, numerous reports have highlighted the vulnerability of histopathologic analysis to error, occasionally including cases with a large specimen from intraoperative resection. Geographically speaking, histopathological diagnosis is more emphasized in Japan, even among marker-positive cases [15], while higher tumor marker levels are routinely considered diagnostically sufficient for treatment in western countries—including enrollment in a variety of clinical trials [16,17]. Accordingly, a true gold-standard diagnostic paradigm integrating tumor markers and histopathological data has not been defined and validated. Newly developed diagnosis methods, including cerebrospinal fluid (CSF) immune cell repertoire [18], copy-number alteration [19], miRNA [20], and detailed imaging investigations [21], have been reported; however, none have been widely integrated, and tumor markers will likely remain a cornerstone of daily clinical practice for physicians managing GCT patients for the foreseeable future.

As GCT is a rare neoplasm that has a geographically distinct distribution worldwide, a straightforward investigation of the relationship between histopathology and tumor markers has not been conducted on a large international cohort. Furthermore, the contemporary moment is potentially optimal for a reassessment of current standard-of-care practices, given the widespread enthusiasm for minimizing any harm from treatment of trial enrollment, as well as individualization of care at diagnostic and therapeutic levels [16,17,22,23].

With these considerations in mind, the primary goal of the current study was to investigate the critical and timely question of whether treating marker-positive GCTs as presumed NGGCTs with malignant components is reliable in a large, multicenter, international cohort study. Additionally, we assessed the ability of needle biopsy to adequately establish definitive diagnosis as compared to tumor markers or microsurgical resection.

## 2. Materials and Methods

The present study is a retrospective cohort study of consecutive, neurosurgically managed, intracranial GCTs treated at the Mayo Clinic during the study period of 1988–2017. This yielded 80 primary cases (not metastatic; not recurrent). The histopathological diagnosis was performed at this institution. All pertinent aspects of the current study were approved and overseen by the institutional review board, including a consent waiver for a minimal-risk study.

The intracranial GCT Genome Analysis Consortium (the iGCT Consortium) database was queried, and 154 primary intracranial GCTs were included for this study. These cases were registered from the neurosurgical departments in Japan participating in the iGCT Consortium. The central histopathological review, according to the WHO classification of tumors of the central nervous system, was performed by a single expert neuropathologist (Y.N.) [2]. The investigation was approved by the ethics committee of the National Cancer Center, Tokyo, Japan, and local institutional review boards.

From these samples, all patients with histopathologic diagnosis and baseline tumor markers were included. Eligible tumor marker results included those measured in serum and/or cerebrospinal fluid (CSF), either during the preoperative period or intraoperatively. The resulting study cohort included 162 cases: 40 from the Mayo Clinic and 122 from the iGCT Consortium.

## 3. Results

### 3.1. Values of Tumor Markers across Histopathology in Tumor Resection Cases

**Figure 1:**
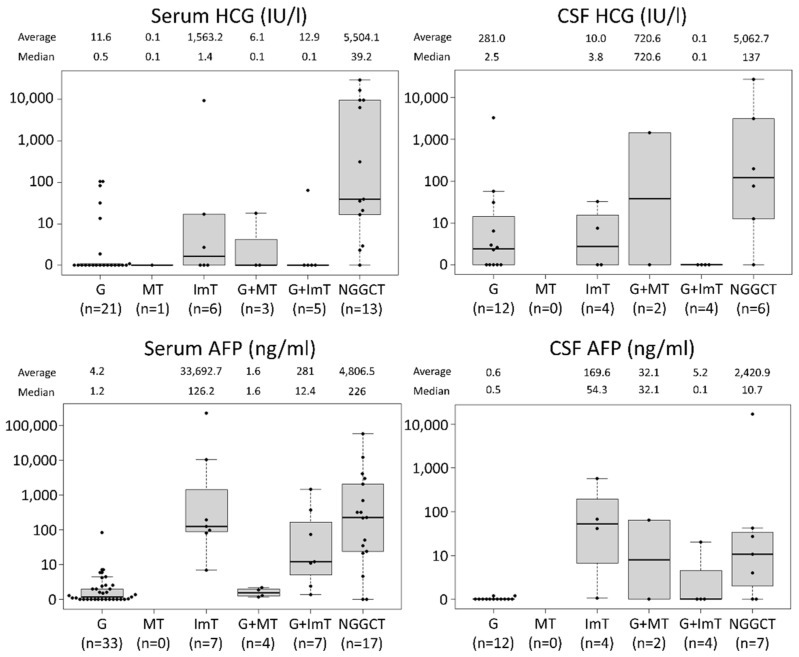
The values for serum and cerebrospinal fluid (CSF) human chorionic gonadotropin (HCG) and serum and CSF alpha fetoprotein (AFP) across histology in 77 cases that underwent resection (not biopsy) are shown. Germinomas were often accompanied by elevated HCG, while immature teratomas were often associated with elevated AFP. Abbreviations; G: germinoma, MT: mature teratoma, ImT: immature teratoma, NGGCT: non-germinomatous germ cell tumor.

There were 77 cases where either one or both of the tumor markers were available and surgical resection was performed (e.g., histopathology specimen volume greater than biopsy from partial, subtotal, or gross total resection). Serum and CSF HCG, and serum and CSF AFP are displayed as scatterplots across histology: germinoma (G), MT, ImT, G + MT, G + ImT, and NGGCTs containing malignant components (YST, CC, EC, and malignant transformation of teratoma) (Figure 1). This shows that NGGCTs generally had exceedingly high values for both HCG and AFP in both serum and CSF. For germinomas, although the median values were frequently within the normal range of HCG (<100 IU/L), some cases demonstrated moderately elevated HCG at or just above the diagnostic threshold. Elevated AFP was only observed in one germinoma case (serum AFP 85 ng/mL), a 4 year-old boy with pineal germinoma, treated with whole-ventricular irradiation and chemotherapy combining cisplatin and etoposide, who had no recurrence during 196 months of follow-up.

ImT was associated with modestly elevated HCG, although the values for all but one case were within the normal range. In contrast, most of the ImT in this cohort showed elevated AFP both in serum and CSF, similar to NGGCTs. This is reflected in the elevated AFP in mixed G + ImT cases.

### 3.2. Distribution of Histology in Marker-Positive, Resected Cases

There were 35 cases (45%) where tumor markers were above the diagnostic threshold (HCG > 100 IU/L or AFP > 10 ng/mL) and upfront resection was performed. The majority of these cases (*n* = 18, 51%) were histopathologically verified as NGGCTs with a malignant component.

**Figure 2:**
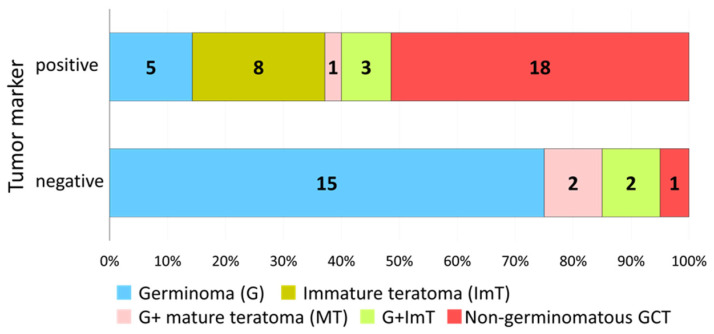
The distribution of the histology in marker-positive (either one or both of the tumor markers in serum or cerebrospinal fluid were positive) and marker-negative cases (both tumor markers were negative) is shown. About half (17 of 35, 49%) of the marker-positive cases did not demonstrate a malignant component on histopathological analysis. Twenty-two cases did not have both tumor markers checked; correspondingly, although the one level that was assessed was negative, these patients were not designated among the definitively marker-negative cases.

Among the other 17 cases (49%), we observed 5 germinomas, 8 ImT, 1 germinoma with MT, and 3 germinomas with ImT (Figure 2). The details of the clinical findings, treatment records, and follow-up are summarized in Table 1. Three of these cases (two ImT and one germinoma) died peri-operatively, two cases (ImT and germinoma with ImT) recurred at 5 and 12 months post-treatment, and one case died (ImT) due to growing teratoma syndrome at 33 months post-treatment. Eleven cases were free from recurrence after cisplatin-containing chemotherapy regimens and irradiation with ventricular coverage (or greater) during follow-up periods of 4–227 months (median 63).

### 3.3. Distribution of Histology in Marker-Negative, Resected Cases

There were 20 cases where tumor markers were negative and upfront resection was carried out. In 19 of 20 such cases (95%), no malignant non-germinomatous histological component was identified (Figure 2). One marker-negative NGGCT case was germinoma with EC.

### 3.4. Distribution of Cases According to HCG and AFP

**Figure 3:**
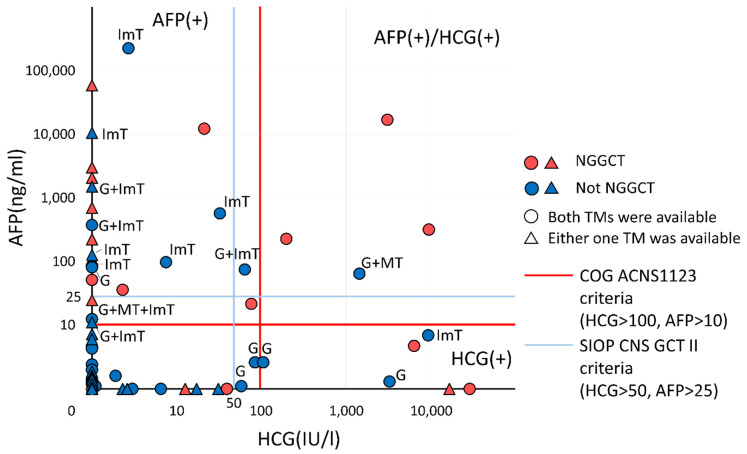
The distribution of human chorionic gonadotropin (HCG) and alpha fetoprotein (AFP) levels are plotted for 77 cases with accessible laboratory data as well as tissue diagnosis from a resection specimen. The red line indicates the criteria in COG ACNS 1123, and the blue line indicates that in SIOP CNS GCT II. No single line completely segregates germ cell tumor (GCT) cases without malignant components. TM: tumor marker, G: germinoma, MT: mature teratoma, ImT: immature teratoma, NGGCT: non-germinomatous GCT.

In total, 77 cases had at least one tumor marker and a surgical resection specimen larger than a biopsy, which were mapped in a tumor marker diagram (Figure 3). Most of the marker-negative cases were not NGGCTs, while the marker-positive cases were typically a mixture of germinoma, MT, and ImT besides NGGCTs. Sensitivity, specificity, and positive/negative predictive values were calculated to better inform clinical application of the study results (Table 2); however, given the limited sample size, a guarded interpretation of these data is recommended.

### 3.5. The Correlation between Tumor Markers and Histopathology in Biopsy Cases

Among 85 biopsy cases, 50 had data for both tumor markers. Within this subgroup, 11 cases (13%) had elevated tumor markers and malignant histopathology; 7 (8%) had negative histopathology in spite of elevated tumor markers; and 32 (38%) had negative tumor markers and negative histopathology. The seven mismatch cases included five germinomas, one germinoma with mature teratoma, and one mature with immature teratoma.

## 4. Discussion

We report the first large, multicenter, international study cohort assessing the utility of tumor markers when combined with histopathologic data—including a unique assessment of the relative diagnostic value of specimens obtained from biopsy as compared to those obtained from resection in establishing a reliable tissue diagnosis. Our data demonstrate compelling evidence that diagnostic accuracy is enhanced by combining histopathologic and laboratory data, as well as confirmation that the potential for sampling error in association with low-volume specimens from biopsy is a source of systematic error likely contributing to misdiagnosis. Correct diagnosis is needed to circumvent under-treatment resulting in the loss of a chance for a cure in NGGCT cases, as well as to avoid administrating toxic treatment to patients if they do not need it.

This study demonstrates that germinomas and teratomas—especially ImT—are associated with elevated tumor markers in the absence of malignant histopathology. In this study, all histopathological specimens were carefully scrutinized, including a central review for cases registered via the iGCT Consortium, and an explicit review by an expert neuropathologist for cases diagnosed at the Mayo Clinic. Correspondingly, our assessment is that the probability of a multiply undiagnosed yolk sac tumor component among the ImT cases with AFP elevation is quite low. HCG elevation was seen in germinoma cases, while AFP elevation was observed in teratomas. HCG is well-known to be a reliable tumor marker for the detection of choriocarcinoma and choriocarcinoma components in mixed GCTs; however, germinoma has also been reported to express HCG at the RNA level, resulting in elevations of the tumor marker in serum and CSF [24,25,26,27]. In this study, only one recurrence was noted among 20 resected germinoma cases, prohibiting a formal analysis of the potential phenotypic differences between marker-positive and marker-negative germinoma. Previous studies indicate that marker-positive germinomas are associated with an unfavorable prognosis compared to marker-negative germinomas [13,24], while HCG-producing germinomas are prognostically comparable to other germinomas [28]. Taken together with the results of the current study, these findings emphasize that germinomas with HCG elevation should not be empirically treated as NGGCTs, given the potential for over-treatment, and individualization of the treatment plan is strongly recommended.

Treatment for ImT has been controversial. Marker-negative, histology-proven ImT is typically regarded as surgically treatable without adjuvant therapies in western countries; however, in Japan, consensus recommendations consider ImT as an intermediate prognostic group, prompting treatment with carboplatin and etoposide chemotherapy, as well as whole-ventricle irradiation. The latter strategy is based on their experience that resection alone or resection with limited irradiation results in a significantly increased risk of recurrence [4]. Additionally, the age of occurrence of ImT may be closely linked with the pathophysiology of the disease; Oosterhuis and Looijenga advocated seven types of whole-body GCTs based on the developmental potentials with distinct genomic and epigenomic features, and categorized ImT and YST arising in patients aged less than 6 years old as a distinct disease subtype from other germinomas and NGGCTs [29]. Interestingly, one case report documents a successful chemotherapeutic cure of such a case arising in an infant [30]. Correspondingly, we concur that ImT in infants and young children, as well as ImT with secreting characteristics, likely warrants intensive therapies; however, treatment strategies for other marker-negative ImTs warrant nuanced consideration.

An important and fundamentally unanswered clinical question raised in the consensus document produced at the 4th International CNS GCT symposium in 2015 was the appropriate cut-off levels for tumor markers [12]. The identification of ideal diagnostic cut-offs is challenging, and both our results and the broader evidence have demonstrated instances of what would constitute over- or under-diagnosis with either the current COG [17] or SIOP [16]. Ultimately, further evidence will be required to better define treatment thresholds and protocols for germinoma and teratoma with secreting characteristics.

A particularly interesting outcome of the current study pertains to the biopsy cases, and the 7-of-50 subgroup in which no malignancy was noted on histopathology in spite of elevated tumor markers. Three of these cases had elevated HCG with germinoma histology, while four of these cases had elevated AFP—only one of which demonstrated ImT on histology (IMT). Given that elevated AFP in the setting of a pure germinoma is essentially unheard of, the more likely explanation of these unusual elevations in AFP are under-diagnosis, referable to the small sample volume obtained via needle biopsy. Table 3 summarizes the interpretation of the correlation of tumor markers and histopathology in biopsy and resection cases individually.

The current study is subject to a range of limitations, including those pertinent to any such study of observational data. Our combined database is heterogeneous, consisting of cases from multiple institutions in Japan and a single US institution, with moderate variability in routine clinical practice and laboratory examinations between institutions. This represents a potential source of confounding or systematic error that could not be adjusted for using the available study data. Additionally, the study focused on a relatively narrow question of the relationship between histopathology and tumor markers, relegating other important clinical perspectives to future analyses. Finally, although the data cleaned from resection are clearly advantageous from a diagnostic perspective, this does not outweigh the additional morbidity of resection-first protocols, and so decision making in many circumstances will be unavoidably limited by the need for reliance on needle biopsy as the preferred method for tissue diagnosis, without a large tumor with symptomatic mass effect. Taken in concert with the study findings, these considerations emphasize the need for a careful and thoughtful interpretation of elevated tumor markers. Diagnostic alternatives, such as miRNA^20^, should be investigated further to enrich the potential of both serum and CSF markers as more highly sensitive and specific diagnostic instruments in the future.

## 5. Conclusions

We report a novel analysis of the relationship between tumor markers and histopathologic findings on either needle biopsy or surgical resection specimens. Our results concern a small but meaningful subpopulation of patients with positive tumor markers who did not have malignant GCT components on histopathology, indicating that some germinomas or teratomas are at increased risk of iatrogenesis if treatment proceeds without formal tissue diagnosis. As such, the question of whether marker-positive GCTs without malignant components in histopathology should be treated as intensively as other NGGCTs remains unanswered—in particular for HCG elevations, which appear to be more common than previously anticipated in non-malignant germinomatous GCTs. In tandem, we also identified cases of significant AFP elevations without malignant components, and we identified confirmatory histopathology on needle biopsy, highlighting the potential limitations of tumor under-sampling as another source of suboptimal treatment for potentially dangerous lesions. In summary, optimizing the diagnostic paradigm for GCTs remains an important area of study, and one that will almost certainly require utilization of both laboratory and pathologic data in order to maximize treatment benefit while minimizing unnecessary risk or harm to a population of young and vulnerable patients.

## Figures and Tables

**Table 1:** Clinical characteristics and follow-ups of 17 cases with positive tumor markers and no malignant non-germinomatous histopathological component, obtained from large resection specimens.

Histopathological Diagnosis	Tumor Location	Age (Years)	Sex	Serum Total HCG (IU/L)	CSF Total HCG (IU/L)	Serum AFP (ng/mL)	CSF AFP (ng/mL)	EOR	Chemotherapy	Radiation Therapy	Recurrence	Alive or Dead	F/U (months)
ImT	Ventricle	0	F			10,481		STR	None	None	No	Dead	0
ImT	Frontal lobe	0	F	2.7		224,865		PR	None	None	No	Dead	0
G	Neurohypophysis + ventricle	24	M		3267.5	1.3	0.8	STR	None	None	No	Dead	0
ImT	Basal ganglia	13	M			126.2	66.9	GTR	PE	WBI + local	No	Alive	4
ImT + G	Pineal	11	M	0.1	0.1	372.8	20.2	GTR	PE	WVI + local	No	Alive	5
ImT	Pineal	16	M	0.1	32.4	192.2	568.59	GTR	PE, ICE, VP16, VBL, TIP	WBI + local	Yes	Dead	5
ImT + G	Pineal	16	M	64		74.5		STR	CARE	WVI	Yes	Dead	12
G	Pineal	25	M	13.4	31			STR	No data	No data	No	Alive	22
ImT	Pineal	11	M	0.1	0.1	80.7	1.06	STR	PE	WBI + local	No	Dead	33
ImT	Pineal	4	M	17.2	0.1			GTR	PE	WVI + local	No	Alive	40
ImT	Pineal	10	M	0.1	7.49	97	41.76	GTR	PE	WBI + local	No	Alive	61
G	Temporal lobe	16	M	105.6		2.6		STR	PE	WBI	No	Alive	63
ImT	Pineal	0	M	9359		6.9		GTR	ICE	CSI + local	No	Alive	86
G + MT	Pineal	14	M	18	1441	2.2	64	STR	ICE	CSI + local	No	Alive	87
G	Pineal	17	M	32	58	1.1	0.1	STR	PE	CSI + local	No	Alive	141
G	Pineal	4	M	0.8		85		GTR	PE	WVI	No	Alive	196
MT + ImT + G	Pineal	12	M	0.1	0.1	12.4	0.1	GTR	PE	No data	No	Alive	227

Abbreviations: CSF; cerebrospinal fluid, EOR; extent of resection, F/U; follow-up, G; germinoma, MT; mature teratoma, ImT; immature teratoma, M; male, F; female, GTR; gross total resection, STR; subtotal resection, PR; partial resection, PE; cisplatin + etoposide, ICE; ifosfamide + cisplatin + etoposide, VBL; vinblastine, TIP; paclitaxel + ifosfamide + cisplatin, CARE; carboplatin + etoposide, WBI; whole-brain irradiation, WVI; whole-ventricular irradiation, CSI; craniospinal irradiation, local; local irradiation.

**Table 2:** Sensitivity, specificity, and positive/negative predictive values of tumor markers for the detection of malignant non-germinomatous germ cell tumor components among cases with tumor resection.

Tumor Markers	Sensitivity	Specificity	PPV	NPV
HCG ≧ 100 IU/L	61.5%	82.1%	53.3%	86.5%
	8/13	32/39	8/15	32/37
AFP ≧ 10 ng/mL	83.3%	78.0%	57.7%	92.9%
	15/18	39/50	15/26	39/42
Either or both	94.7%	52.8%	51.4%	95.0%
	18/19	19/36	18/35	19/20

Abbreviations: HCG; human chorionic gonadotropin, AFP; alpha fetoprotein, PPV; positive predictive value, NPV; negative predictive value.

**Table 3:** Interpretation of the correlation of tumor markers and histopathology in biopsy and resection cases. For biopsy cases, 50 of 85 cases with both tumor markers available were included. For resection cases, 22 of 77 cases with only one tumor marker checked were not included.

**Biopsy Cases**			
Tumor marker (TM)	Malignancy in histopathology	Number of cases	Interpretation
(+)	(+)	11	TM was right. Biopsy was not mandatory.
(+)	(−)	7	Showing limitation in histopathology by biopsy specimen.
(−)	(+)	0	Not applicable.
(−)	(−)	32	TM and histopathology were in accordance.
**Resection Cases**			
Tumor marker (TM)	Malignancy in histopathology	Number of cases	Interpretation
(+)	(+)	18	TM was right. Resection was not mandatory.
(+)	(−)	17	Limitation in TMs. Possibility of over-treatment.
(−)	(+)	1	Corroborates biopsy in TM-negative cases.
(−)	(−)	19	TM and histopathology were in accordance.

## Data Availability

The data presented in this study are available on request from the corresponding author.
